# CT-based 3D-printed coronary artery phantom for imaging projection education

**DOI:** 10.2478/raon-2026-0027

**Published:** 2026-05-14

**Authors:** Laura Jursa, Masa Kramer, Anja Boc, Nejc Mekis

**Affiliations:** 1 Medical imaging and radiotherapy department, Faculty of Health Sciences, University of Ljubljana, Ljubljana, Slovenia; 2 Institute of Anatomy, Faculty of Medicine, University of Ljubljana, Ljubljana, Slovenia

**Keywords:** three-dimensional printing, invasive coronary angiography, coronary arteries, X-ray phantom

## Abstract

**Background:**

Coronary artery disease is among the leading causes of death worldwide. The method of choice for coronary artery imaging is catheterization using real-time fluoroscopic images of coronary anatomy and pathology. The aim of the study was to create an anatomically accurate cardiac and coronary artery phantom suitable for learning and practicing image projections in invasive coronary angiography by using computed tomography (CT) image segmentation and 3D printing.

**Materials and methods:**

The development of an anatomically accurate heart phantom was carried out in several phases. In the first phase, the material for 3D printing was analysed and the best material with a similar attenuation coefficient to the heart muscle was selected. Then the segmentation of the heart was performed with the 3D Slicer software based on the CT scan. Afterward the phantom of the heart was developed. The anatomy professor of the medical faculty drew coronary arteries on the phantom. The mixture of metal powder (tungsten) and ultraviolet (UV) gel was used to draw the coronary arteries on the heart phantom. In the final stage, the phantom was verified by obtaining and comparing fluoroscopic images of the phantom to reference coronary angiograms from the literature.

**Results:**

When comparing the images of the phantom with the images from the literature, we found that the phantom represents an anatomically correct course of the left and right coronary arteries.

**Conclusions:**

It can be concluded that the 3D printing technique can be used to develop an anatomically correct heart phantom with coronary arteries that can be used as a teaching tool for radiographers in fluoroscopic visualization of coronary arteries. Consequently, significantly contributing to a faster and safer practise.

## Introduction

Coronary artery disease (CAD) is the third leading cause of death, responsible for approximately 9.1 million deaths worldwide each year.^1^ The most common cause of CAD is atherosclerosis^2^ leaving the accumulation of plaque within the arterial wall. The progressive narrowing of one or more coronary arteries can lead to acute myocardial infarction or sudden cardiac death.^3-5^ In patients at high risk of CAD, invasive coronary angiography remains the primary imaging modality, providing real-time fluoroscopic visualisation of coronary anatomy and pathology.^6,7^ In case of detected clinically significant stenosis, the procedure can be immediately extended into a therapeutic intervention^7^, most commonly involving balloon dilatation followed by stent placement.^6,8^ As with other X-ray techniques, coronary angiography requires two perpendicular views to ensure accurate assessment of the coronary arteries.^7^

Due to anatomic variants and pathologic changes, the shape and course of the coronary arteries may vary from patient to patient. The projections listed in the literature can serve as guidelines but often need to be adjusted based on the vessel segment and the patient’s individual anatomy.^7,9^ Two main projections commonly used in coronary angiography are the right anterior oblique projection (RAO) and the left anterior oblique projection (LAO). They are named according to the position of the detector in relation to the patient. These projections can be obtained at different angles depending on the inclination of the detector. In addition, the C-arm can be angled cranially or caudally to optimize the visualization of certain segments.^7^ Therefore, a thorough understanding of vascular anatomy and fluoroscopic projection techniques is crucial for the proper performance of the procedure.7,9 This knowledge can be acquired through the use of phantoms, especially anthropomorphic phantoms that simulate the anatomical structures and attenuation characteristics of human tissue.^10^ However, due to high production costs, such phantoms are often expensive, which has a negative impact on their accessibility, making them unavailable especially in low-income countries where finances and human resources are limited.^11^ As a result, there is growing interest in more cost-effective production alternatives.

Recent technological advances in Three-Dimensional (3D) printing have made it a promising method for the production of specific, anatomically accurate phantoms representing different organ systems.^12,13^ Fused Deposition Modelling (FDM) and Stereolithography (SLA) are the predominant methods in the production of phantoms.^10^ The biggest advantage of 3D printing is its low cost. Once the system is set up, it only costs about $500 to produce the phantom, as opposed to standard X-ray phantoms, which can cost more than $20,000. This makes 3D printed phantoms more affordable for educational and other purposes.^14^ Attenuation properties similar to those of human tissue can be achieved by using different printing materials such as polylactic acid (PLA), acrylonitrile butadiene styrene (ABS) and SLA resin, either alone or in combination with certain materials.^10,15^ Attenuation properties can also be altered by moderating infill ratios and patterns.^10^ The aim of the study was to create an anatomically accurate cardiac and coronary artery phantom suitable for learning and practicing image projections in invasive coronary angiography by using computed tomography (CT) image segmentation and 3D printing.

## Materials and methods

The study comprised the following four steps: material analysis, model design, phantom fabrication, and verification.

### Material analysis

For the heart phantom, Five different filament materials were tested: PLA, PLA Wood, PLAbio, polycarbonate and nylon. The filaments were attached to an AGFA DX-D 40 detector (AGFA, Belgium) and imaged with a Siemens Multix/Vertix unit (Siemens, Germany). Based on the results, PLA was deemed most suitable due to its attenuation properties and low toxicity.

For the coronary artery phantom, we investigated ultraviolet (UV) nail gel (Semilac, Poland) mixed with metal powder, which has a high tungsten content. The mixture was applied on an acrylic base and cured with a UV/LED lamp SUN x 16 MAX (Guangzhou Yokefellow Electronic Co., Ltd, China) with 72 LED lamps and a power of 320 W. We tested three different ratios of UV gel to powder: 1:0.5, 1:1, and 1:1.5, and chose the 1:1.5 ratio for the radiographic evaluation due to its high radiopacity. Tungsten was chosen mainly because of its high atomic number (74) and its attenuation properties.^16^

### Model design

The phantom was designed using a chest CT scan that was part of the COVID-19-AR series.^17^ Data were obtained from The Cancer Imaging Archive (TCIA) website, which provides access to anonymized medical images. Image segmentation was performed using 3D Slicer software (v 5.6.2).^18^

We created two separate segments, the background and the heart. The latter included the entire heart and the proximal part of the ascending aorta, excluding other vessels to simplify the model and reduce printing time. We marked the heart and the background on every third layer with the painting tool. Then we performed segmentation using the semi-automatic ‘grow from seeds’ method, which predicts the shape of the structure based on the marked areas. The obtained model (Model A) was exported and saved. The segmentation was further processed to create the second (Model B), and the third model (Model C). For Model B, we applied the smoothing algorithms such as Gaussian and median filters. The model was hollowed out, leaving the wall thickness of 7.8 mm. In Model C, we reduced the wall thickness to 3.9 mm. All segmentations were exported to FlashPrint Software v 5.8.7 (Zhejiang Flashforge 3D Technology Co., Ltd., China) to determine the models’ infill patterns and ratios. In Model A, the honeycomb filling pattern was evenly distributed throughout the entire volume. In Model B, the pattern was present in the wall, while the rest of the model remained hollow. In model C, the inner and outer walls had 6 layers and an infill of 100% filling, while the rest (the core) remained hollow. To achieve an anatomically correct position of the model, we designed a customized base in 3D Builder software (Microsoft, USA).

To ensure a perfect position of the model during X-ray imaging, the base was made as a negative of the 3D heart model.

### Phantom fabrication

All models were produced with the Flashforge Guider 3 3D printer (Zhejiang Flashforge 3D Technology Co., Ltd., China) using the FDM process and white PLA filament. The print nozzle was set to 210°C, and the print bed to 50°C. Model A took approximately 12 hours to print, Model B 16 hours, and model C 11 hours. Models B and C were printed as two separate halves to achieve a hollow core. The two parts of models B and C were then glued together with a modelling adhesive. During X-ray imaging, Model C was the most suitable as it showed hardly any artefacts. Therefore, two additional samples of Model C were printed.

The base was printed with the smallest achievable PLA layer thickness (1mm) to minimize artefacts.

An anatomist illustrated the following coronary arteries on the samples of Model C: the left main coronary artery (LM), the left anterior descending artery (LAD) with its diagonal (D) branches, the left circumflex artery (LCX) with its marginal (M) branches, and the right coronary artery (RCA) with its two branches, the acute marginal artery (AM) and the posterior descending artery (PDA). The left and right coronary arteries with their branches were illustrated on the separate model samples.

### Phantom verification

Fluoroscopic imaging was performed at the University Medical Centre Ljubljana using a Siemens Artis Q C-arm (Siemens, Germany). A total of nine fluoroscopic images were taken in different projections. Three for the right and six for the left coronary artery. Images were acquired according to the department’s clinical protocol (Floro-Cardio 7.5 p/s, 81 kV, 4 mAs). To evaluate anatomic accuracy and arterial visibility, the obtained images were compared with the reference coronary angiograms from the IMAIOS website.^19^ Table 1 presents the projections used in fluoroscopic imaging of the phantoms as well as projections used for the reference images.

**TABLE 1.** Projections used in fluoroscopic imaging of the heart phantom with coronary arteries and projections from the literature used for comparison with our images^19^

	Projections used in phantom imaging	Projections from the literature
**Left coronary Artery**	LAO 45° – CAU 40°	LAO 30° – CAU 30°
	LAO 35° – CRA 30°	LAO 45° – CRA 20°
	AP 0° – CRA 35°	LAO 0° – CRA 30°
	RAO 40° – CRA 35°	RAO 30° – CRA 30°
	RAO 40° – CAU 30°	
	AP 0° – CAU 40°	RAO 0° – CAU 30°
**Right coronary Artery**	LAO 40°	LAO 45° – CAU 15°
	AP 0° – CRA 30°	
	RAO 30°	LAO 45° – CAU 15°

1 AP = anteroposterior; CAU = Caudal; CRA = Cranial; LAO = Left Anterior Oblique; RAO = Right Anterior Oblique

## Results

### Phantom design and fabrication

**Figure 1.**
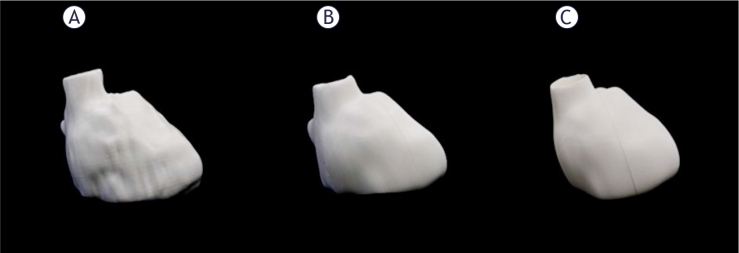
3D-printed model with honeycomb shaped infill **(A)** hollow model with honeycomb shaped infill of the walls **(B)**, and hollow model with 100% infill of the walls **(C)**.

Three different models were printed using PLA material and the FDM printing technique, as shown in Figure 1. Model A has an uneven surface due to the lack of smoothing filters. Models B and C have a smoother surface due to the application of filters, and are both hollow: the wall thickness in model B is 7.8 mm, and in model C 3.9 mm.

**FIGURE 2.**
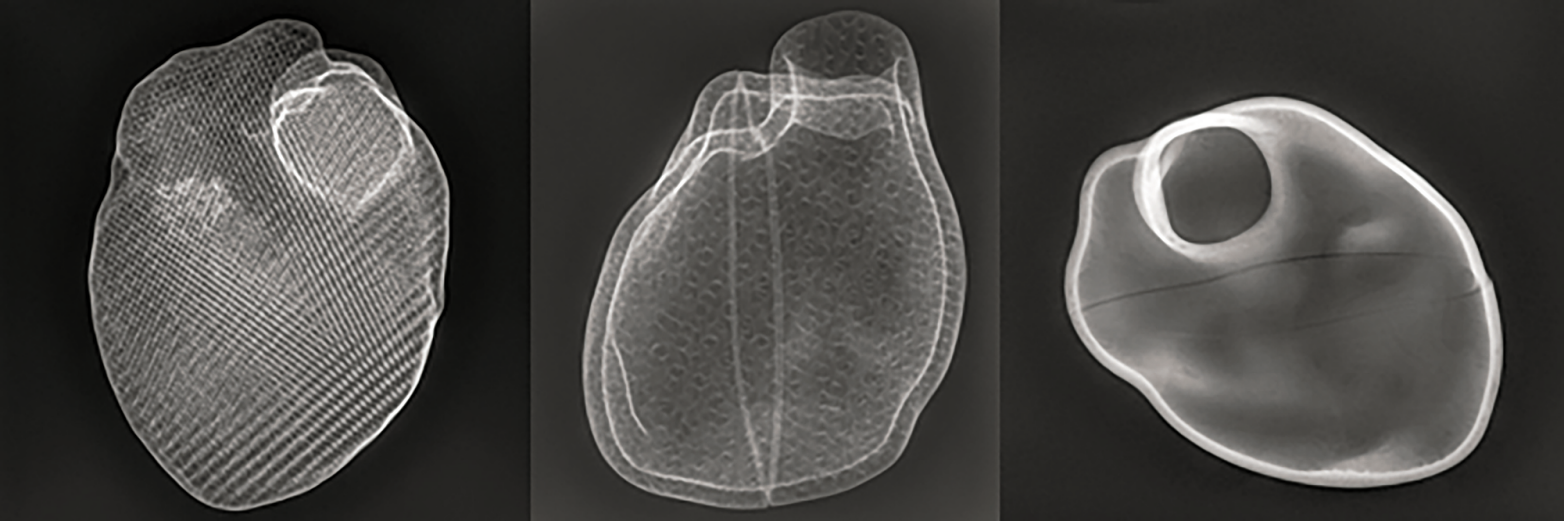
Radiographs of 3 different heat phantoms: full model with honeycomb shaped infill **(A)**, hollow model with honeycomb shaped infill of the walls **(B)**, and hollow model with 100% infill of the walls **(C)**.

The radiographic images of all three models are presented in Figure 2. A distinct honeycomb artefact is visible in Model A which was produced with a honeycomb filling evenly distributed over the entire volume. Although hollowed, Model B contained a honeycomb pattern in its walls, therefore the honeycomb artefact was still on the radiograph, but was less pronounced than in Model A.

Additionally, white line on Figure 2(B) was present on the radiograph of Model B at the site where the two parts of model were glued together. In Model C, the glued junction between the two parts can be seen as a thin dark line. The walls were build with up to twelve layers and an infill of 100%, while the rest of the model remained hollow. No significant artifacts were visible on radiograph of model C, with the exception of some contours representing the cardiac wall.

**FIGURE 3.**
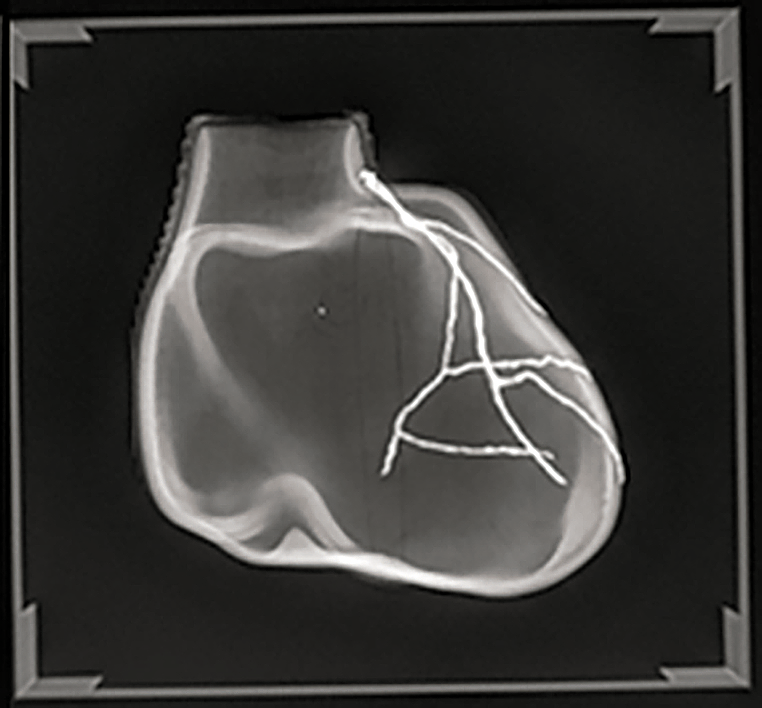
Radiograph of the heart model with vessels drawn by a mixture of UV gel and metallic powder.

Figure 3 shows a radiograph of Model C with drawn coronary arteries, acquired with general X-ray unit and placed in the custom base to ensure a similar position of the heart as in the patient

**Figure 4.**
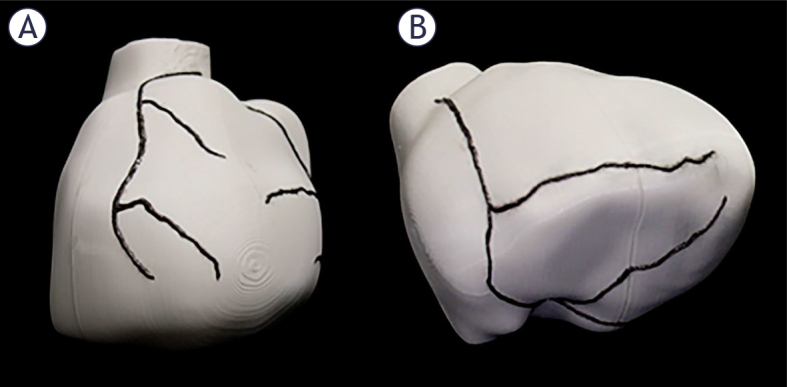
3D-printed heart model with the left **(A)**, and the right **(B)** coronary artery.

**Figure 5.**
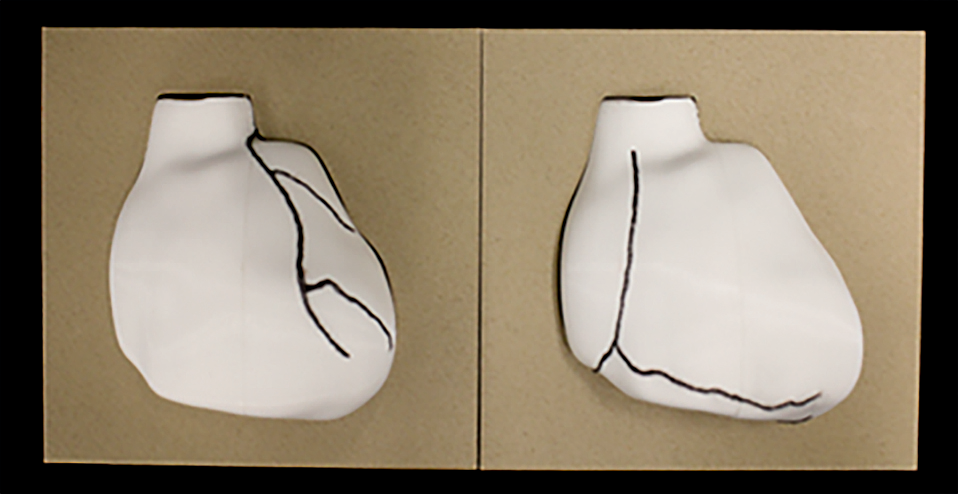
3D-printed models in their custom bases.

Using the Model C technique, two heart phantoms were created. On each model, one coronary artery with its branches was drawn (Figure 4). Figure 5 shows the two phantoms in their customized bases.

### Phantom verification

As mentioned in methods acquired fluoroscopic images were compared with those obtained from the IMAIOS website.

Table 2 presents the comparison between the obtained and the reference images for RCA, AM and PDA. LAO 40° clearly shows the proximal and mid segments of the RCA. On the reference image (LAO 45°, CAU 15°), the RCA appears more C-shaped. In AP 0°, CRA 30°, the initial segment of the mid RCA is clearly recognizable, while the distal PDA is poorly defined. A comparison was not possible as there are only a few reference images in the literature. RAO 30° clearly shows the RCA over its entire length, including the PDA. A similar arterial course can also be seen on the reference image (RAO 30°).

**TABLE 2.** Comparison of the obtained right coronary artery images of the heart model with images from the literature

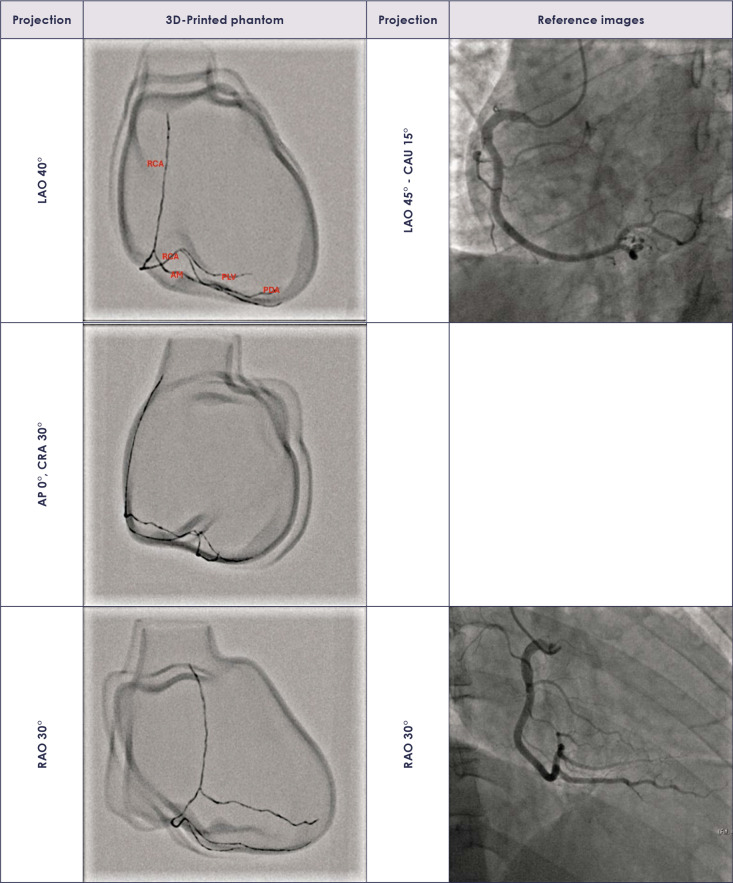

1 AP = anteroposterior; CAU = Caudal; CRA = Cranial; LAO = Left Anterior Oblique; RAO = Right Anterior Oblique

Table 3 presents the comparison between the obtained and the reference images for LM, LAD and LCX. LAO 45°, CAU 40° shows the bifurcation of LM in LAD and LCX, together with their marginal (M1, M2) and diagonal (D1, D2) branches. A similar but slightly different course can be seen on the reference image (LAO 30°, CAU 30°). LAO 35°, CRA 30° clearly shows the proximal and mid LAD and LCX. A different coronary path is observed in the reference image (LAO 45°, CRA 20°) due to the different angles. AP 0°, CRA 35° provides a good view of the distal LAD and its diagonal branches, while the LCX is poorly visible. A similar vessel course is observed in the reference image (AP 0°, CRA 30°). At RAO 40°, CRA 35°, the mid, distal LAD and the diagonal branches are clearly visible, but the image does not match the reference image (RAO 30°, CRA 30°). RAO 40°, CAU 30° shows the LM bifurcation, the proximal LAD and LCX as well as the marginal branches, while the diagonal branches are poorly visible due to superimposition. Due to the limited number of reference images, no comparison was performed. In AP 0°, CAU 40° the LM, the distal LAD, the LCX and the marginal branches are clearly visible. Due to the different angulation, the image does not match the reference image (RAO 30°, CRA 30°).

**TABLE 3.** Comparison of the obtained right coronary artery images of the heart model with images from the literature

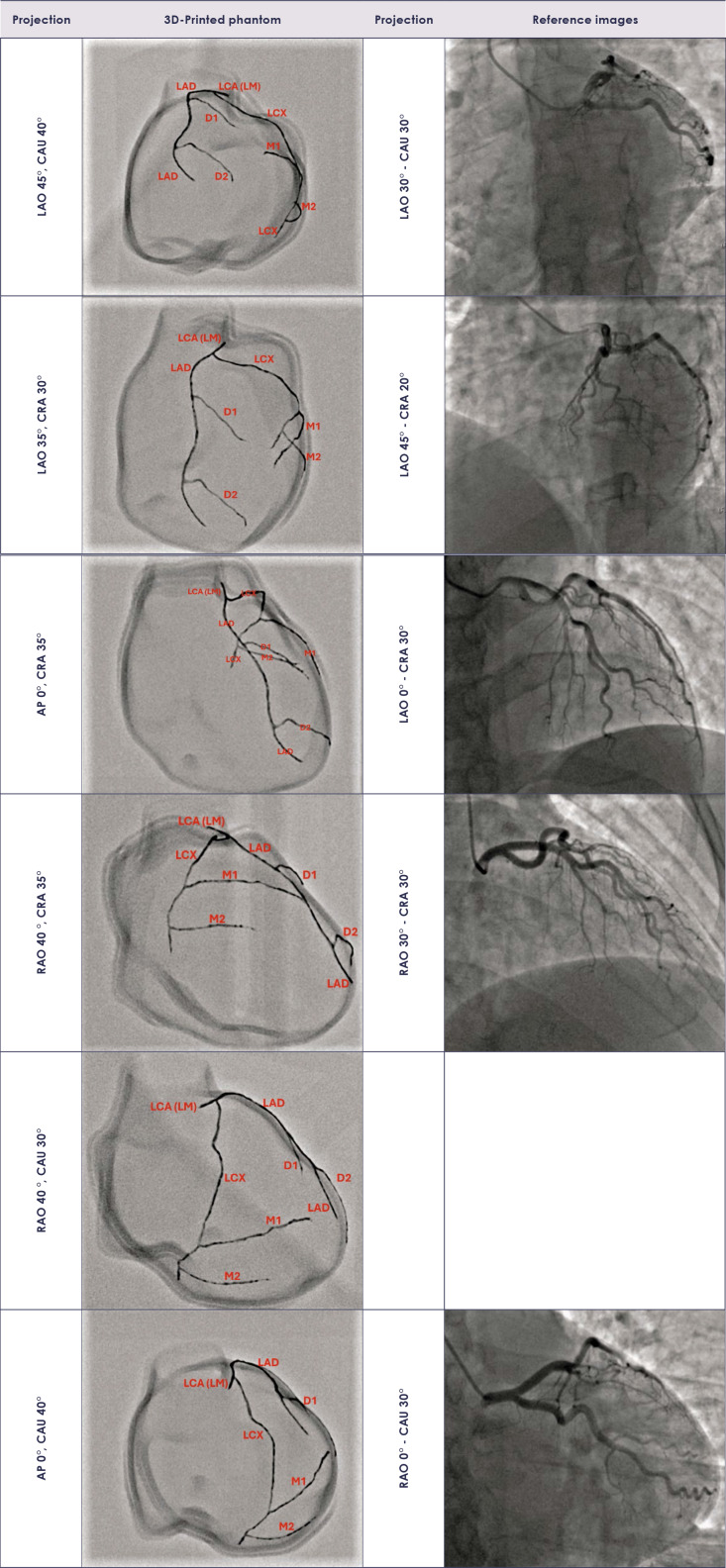

1 ^1^ RAO = Right anterior oblique, LAO = Left anterior oblique, AP = Anteroposterior, CRA = Cranial, CAU = Caudal.

1 AP = anteroposterior; CAU = Caudal; CRA = Cranial; LAO = Left Anterior Oblique; RAO = Right Anterior Oblique

## Discussion

The aim of the study was to develop an anatomically accurate X-ray phantom of the heart with coronary arteries for use in education and training in invasive coronary angiography, using the CT image segmentation and 3D printing. In the end, two phantoms were created, one for each coronary artery along with two costume bases that allow for anatomically correct positioning of the phantoms.

In similar studies, PLA and ABS were the most commonly used materials to mimic soft tissues such as fat and glandular tissue.^20^ Both have attenuation properties similar to those of water and can therefore be used as tissue substitutes. Their costs are also similar.^14,21^ However, during the printing process, ABS emits styrene, which can be potentially harmful if adequate ventilation is not provided.^22^ This was the main reason for excluding ABS from the materials tested in our study. Based on the imaging results, PLA was selected as the most suitable among tested materials due to its attenuation properties, low toxicity and cost-effectiveness. The attenuation properties of PLA lie between those of water and bone^14^, with a linear attenuation coefficient very similar to that of muscle tissue (0.7773–0.1996 cm^-1^ and 0.756–0.154 cm^-1^ for 20–100 kV, respectively).^23,24^ Density similarities were also confirmed; PLA has a density of approximately 1.24 g/cm^3^,^25^ while the density of cardiac muscle has recently been reported to be 1.055-1.265 g/cm^3^.^26^

Several studies have reported that the attenuation properties and thus the presence of artifacts can be influenced by changing the infill pattern and ratio.^10,27^ Similar results were observed in our study, where we tested three models, two with the use of a honeycomb pattern (one full and one hollow) and one with the use of 100% infill. The use of honeycomb pattern produced distinct artefacts, while almost no artefacts were observed at 100% infill. This is probably due to the different attenuation of the beam with the different infill patterns. When the beam passed through the 100% infill, the attenuation was uniform. In contrast, the honeycomb pattern caused uneven attenuation, resulting in artefacts that resembled the original grid-like pattern. Even with the 100% infill some contours of the cardiac wall were still visible due to the plastic superimposition, but these did not affect the image quality and did not obscure the arteries.

Both printed phantoms with drawn coronary arteries accurately represented the anatomical position and course of the arteries in relation to the heart wall, which is visible on both the physical models and the X-ray images. This proves that an anatomically accurate X-ray phantom can be produced using the CT image segmentation and 3D printing. A major advantage of this phantom is the relative cost. Once the system is established, the material costs around €15 per phantom. This makes the model accessible to a larger population group and thus contributes to patient safety.

The study also aimed to produce images comparable to real coronary angiograms. This was generally achieved as the main anatomical features of the coronary arteries in our models matched the reference images. However, there were some inconsistencies between the obtained and the reference X-ray images. The main reason was the use of different imaging protocols, with projection angles differing by up to 15°, which significantly affected structural alignment. Additionally, anatomical differences in the location of the coronary arteries in each patient may result in the phantom arteries not fully matching the reference images. Nevertheless, the X-ray images of our models were comparable to real coronary angiograms when similar projection angles were used.

Our study has several limitations. Since the models were based on CT segmentation, the surface relief of the heart was not well defined. The coronary and interventricular sulci were further blurred by the filters applied during segmentation, which may have affected the accuracy of coronary drawing placement. Interventricular septum was not printed; therefore, septal branches of LAD and PDA are missing. The diameters of different arteries were not taken into account, as the thickness of the UV gel-powder mixture prevented precise delineation.
